# Development of a scoring model based on objective factors to predict gangrenous/perforated appendicitis

**DOI:** 10.1186/s12876-023-02767-7

**Published:** 2023-06-07

**Authors:** Toshimichi Kobayashi, Eiji Hidaka, Itsuki Koganezawa, Masashi Nakagawa, Kei Yokozuka, Shigeto Ochiai, Takahiro Gunji, Toru Sano, Koichi Tomita, Satoshi Tabuchi, Naokazu Chiba, Shigeyuki Kawachi

**Affiliations:** Department of Digestive and Transplantation Surgery, Tokyo Medical University, Hachioji Medical Center, 1163 Tatemachi Hachioji, Tokyo, 193-0998 Japan

**Keywords:** Gangrenous/perforated appendicitis, Scoring model, Prediction

## Abstract

**Background:**

The mortality rate of gangrenous/perforated appendicitis is higher than that of uncomplicated appendicitis. However, non-operative management of such patients is ineffective. This necessitates their careful exam at presentation to identify gangrenous/perforated appendicitis and aid surgical decision-making. Therefore, this study aimed to develop a new scoring model based on objective findings to predict gangrenous/perforated appendicitis in adults.

**Methods:**

We retrospectively analyzed 151 patients with acute appendicitis who underwent emergency surgery between January 2014 and June 2021. We performed univariate and multivariate analyses to identify independent objective predictors of gangrenous/perforated appendicitis, and a new scoring model was developed based on logistic regression coefficients for independent predictors. Receiver operating characteristic (ROC) curve analysis and the Hosmer–Lemeshow test were performed to assess the discrimination and calibration of the model. Finally, the scores were classified into three categories based on the probability of gangrenous/perforated appendicitis.

**Results:**

Among the 151 patients, 85 and 66 patients were diagnosed with gangrenous/perforated appendicitis and uncomplicated appendicitis, respectively. Using the multivariate analysis, C-reactive protein level, maximal outer diameter of the appendix, and presence of appendiceal fecalith were identified as independent predictors for developing gangrenous/perforated appendicitis. Our novel scoring model was developed based on three independent predictors and ranged from 0 to 3. The area under the ROC curve was 0.792 (95% confidence interval, 0.721—0.863), and the Hosmer–Lemeshow test showed a good calibration of the novel scoring model (*P* = 0.716). Three risk categories were classified: low, moderate, and high risk with probabilities of 30.9%, 63.8%, and 94.4%, respectively.

**Conclusions:**

Our scoring model can objectively and reproducibly identify gangrenous/perforated appendicitis with good diagnostic accuracy and help in determining the degree of urgency and in making decisions about appendicitis management.

## Background

Acute appendicitis is the commonest cause of acute abdomen, and the lifetime prevalence of appendicitis is approximately 7–8% [1]. Acute appendicitis is categorized as uncomplicated or complicated; approximately 30% of cases of acute appendicitis in the United States are considered complicated [2]. Acute complicated appendicitis is commonly defined as appendiceal inflammation with signs of gangrene, perforation, or abscess. It may eventually lead to acute diffuse peritonitis associated with a high risk of morbidity and mortality. The mortality rate is higher in gangrenous appendicitis (0.6%) than in uncomplicated appendicitis (UA) (< 0.1%). Furthermore, the mortality rate of appendicitis is higher in perforated appendicitis (5%) than in uncomplicated appendicitis [3].

Recent studies have reported the efficacy and safety of non-operative management using antibiotic therapy for UA [4, 5]. In addition, it is easier to diagnose acute appendicitis with an abscess using computed tomography (CT), and non-operative management with interval appendectomy is safe and effective [6–8]. Recently, in our department, interval appendectomy following non-operative management has become the first choice of management in treating appendicitis with abscess. However, some studies have reported that non-operative management for gangrenous/perforated appendicitis (GPA) is unlikely to be effective, and surgical intervention is frequently required [1, 9, 10]. Recent studies have reported that failure of non-operative management in GPA was associated with an increased need for open surgery, major bowel resection, and prolonged length of hospital stay [11, 12]. Thus, to improve the prognosis of patients with appendicitis, a thorough examination of patients with GPA at presentation is of great clinical significance and can help surgeons to make an urgent decision on surgical management of the patient as opposed to patients with UA. However, identifying GPA is difficult for surgeons and remains challenging. Scoring models to predict GPA without the evidence of an abscess have been reported by few studies. Moreover, scoring models to predict complicated acute appendicitis have been reported by recent studies [13–18]; however, most of the models included variables with poor objectivity and reproducibility, such as symptoms and physical examination. Therefore, in this study, we aimed to develop a novel scoring model based on objective findings such as patient characteristics and serological and radiological findings in predicting GPA (excluding appendicitis with an abscess).

## Methods

### Patients

We retrospectively analyzed 202 patients (aged > 16 years) who were diagnosed with acute appendicitis based on clinical symptoms and CT findings and underwent emergency surgery between January 2014 and June 2021 at our institution. In our department, there are no established criteria for treatment selection, rather it is largely at the discretion of the attending physician. Recently, however, appendicitis with mild inflammation is often treated nonoperatively. Gangrenous appendicitis was diagnosed pathologically, whereas perforated appendicitis was diagnosed either pathologically or based on surgical findings, such as a perforated appendix, or based on CT findings, such as the presence of air outside the lumen. Among the 202 patients included in this study, patients with an abdominal abscess confirmed by CT (n = 44), appendiceal neoplasm, malignancy (n = 3), and missing data (n = 4) were excluded. A total of 151 patients were thus ultimately enrolled in this study and were categorized into two groups, namely, GPA and UA groups.

This study was approved by the Tokyo Medical University Hachioji Medical Center Ethics Committee (approval no. T2020-0314). Informed consent was obtained in the form of an opt-out.

### Study variables

Patient information, laboratory, and radiological findings were extracted from electronic medical records. Patient characteristics and preoperative variables included sex; age; body mass index; a past medical history of appendicitis; a past medical history of abdominal surgery; and laboratory findings, including white blood cell count, C-reactive protein (CRP), platelets, bilirubin, and creatinine. Radiographic findings on CT imaging included the maximal outer diameter of the appendix (in mm), the presence of appendiceal fecalith, the presence of periappendiceal fat stranding, and the presence of free intraperitoneal fluid. All the above-mentioned variables are highly objective.

### Statistical analyses

We used the Statistical Package for Social Sciences version 27.00 (IBM Corp, Armonk, NY, USA) for all statistical analyses. Univariate analysis was used to compare patient demographics, preoperative, and radiographic variables between the GPA and the UA groups. The Mann–Whitney U test was used for the comparison of continuous variables, and Fisher’s exact test or the chi-squared test was used for the comparison of categorical variables. *P < 0.05* was considered significant. Multivariate logistic regression analysis was used for patient demographics, preoperative, and radiographic variables with *P < 0.05*.

All variables with *P < 0.05* in the multivariate analysis were identified as independent predictors of GPA and were used for the final model. Continuous predictor variables were converted to binary variables based on cut-off values set by using the receiver operating characteristic (ROC) curve analysis.

We developed a new scoring model based on logistic regression coefficients for independent predictors. For the scoring model, ROC curve analysis was used to evaluate discrimination, and the Hosmer–Lemeshow test was used for calibration. The area under the ROC curve (AUC) is a measure of the accuracy of a quantitative diagnostic test. The Hosmer–Lemeshow test is a statistical test to determine goodness-of-fit, where *P > 0.05* indicates adequate calibration. Finally, scoring was classified into three categories, and the probability of GPA in each category was assessed. We estimated the power to compare the score of the model between GPA and UA groups using a post hoc power analysis of the two-tailed independent t-test at 5% alpha.

## Results

Of the 151 patients included in this study, the median age was 47 (range, 16–89) years, and 97 (64.2%) patients were male. We categorized 85 (56.3%) patients and 66 (43.7%) patients into the GPA and UA groups, respectively. None of the patients had undergone preoperative percutaneous drainage.

### Comparisons between GPA and UA groups using univariate and multivariate analyses

Univariate analysis was performed to compare the GPA and UA groups. Patient characteristics and preoperative variables of both groups are shown in Table 1. A significant difference was found only in age with respect to patient characteristics, where patients in the GPA group were older (*P = 0.006*) than those in the UA group. Regarding laboratory and CT findings, CRP (*P* < 0.001), bilirubin (*P* = 0.025), and the maximal outer diameter of the appendix (*P* < 0.001) were significantly higher in the GPA group than in the UA group. Additionally, more patients had fecaliths (*P* < 0.001) and periappendiceal fat stranding (*P* < 0.001) in the GPA group than in the UA group.

**Table 1:** Comparison Between the GPA-Group and UA-Group of Patients Characteristics and Preoperative Variables

	GPA-group (n = 85)	UA-group (n = 66)	*P* value
**Patient characteristics**			
Age	52(16–89)	41(16–81)	0.006
Male sex	57(67.1%)	40(60.6%)	0.412
Body mass index (kg/m^2^)	22.6(17.2–32.7)	22.5(15.8–33.1)	0.723
Previous history of appendicitis	6(7.1%)	6(9.1%)	0.647
Previous history of abdominal surgery	12(14.1%)	6(9.1%)	0.344
**Laboratory findings**			
WBC (/µL)	14,500(2270–43,500)	12,850(4400–25,700)	0.069
CRP (mg/dL)	11.02(0.02–54.88)	1.83(0.02–27.50)	< 0.001
Plt (×10^4^/µL)	21.5(9.1–35)	22.6(9.6–41.5)	0.37
Bil (mg/dL)	1.1(0.2–5.1)	0.95(0.3–2.4)	0.025
Cre (mg/dL)	0.7(0.37–10.40)	0.69(0.30-10.45)	0.794
**CT findings**			
Maximal diameter of the appendix (mm)	13(7–19)	11.5(6–19)	< 0.001
Presence of fecalith	68(80%)	34(51.5%)	< 0.001
Presence of periappendiceal fat stranding	73(85.9%)	38(57.6%)	< 0.001
Presence of free peritoneal fluid	16(18.8%)	6(9.1%)	0.072

Categorical data are expressed as percentages and continuous data are expressed as median (min-max)

WBC, white blood cell; CRP, C-reactive protein; Plt, platelet; Bil, bilirubin; Cre, creatinine; CT, computed tomography, GPA-group, gangrenous/perforated appendicitis group; UA-group, uncomplicated appendicitis group

We performed multivariate logistic regression analysis for six variables (age, CRP, bilirubin, maximal outer diameter of the appendix, presence of appendiceal fecalith, and presence of periappendiceal fat stranding) which showed that CRP, the maximal outer diameter of the appendix, and the presence of appendiceal fecalith were independent predictors of GPA (Table 2).

**Table 2:** Multivariable Logistic Regression Analysis of Predictors for Gangrenous/Perforated Appendicitis

Variables	OR (95% CI)	*P* value
Age	–	–
CRP	1.111 (1.055–1.17)	< 0.001
Bilirubin	–	–
Maximal diameter of the appendix(mm)	1.176 (1.014–1.364)	0.032
Presence of fecalith	2.965 (1.325–6.636)	0.008
Presence of periappendiceal fat stranding	–	–

CRP, C-reactive protein; OR, odds ratio; CI, confidence interval

### Development of a scoring model for the prediction of GPA

We developed a scoring model for predicting GPA based on the final logistic regression model. Continuous variables (CRP and the maximal outer diameter of the appendix) were converted to a binary variable according to the optimal cut-off value with the highest sum of sensitivity and specificity. The cut-off value was set to 7 mg/dL for CRP and 13 mm for the maximal outer diameter of the appendix. For convenience, the regression coefficients assigned to each predictor were rounded down to the nearest integer, and the sum of the scores assigned to each predictor was considered the total score of the new scoring model and ranged from 0 to 3 (Table 3).

**Table 3:** Scoring Model of Prediction for GPA

Variables	Regression coefficient	Score
CRP ≥ 7 (mg/dL)	1.73	1
Maximal outer diameter of the appendix ≥ 13 (mm)	1.129	1
Presence of appendiceal fecalith	1.137	1
Total		0–3

GPA, gangrenous/perforated appendicitis; CRP, C-reactive protein

**Fig. 1:**
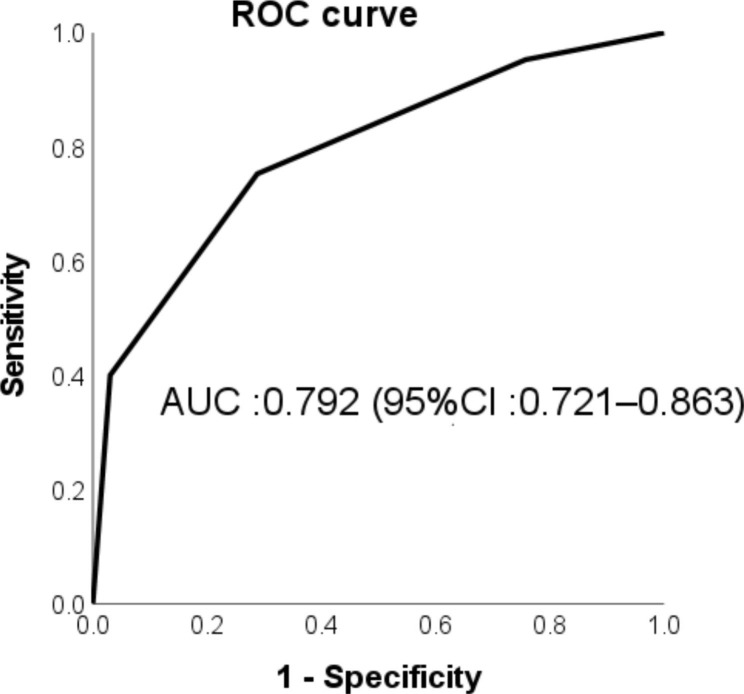
Receiver operating characteristic (ROC) curve for the new scoring model. The area under the ROC curve (AUC) for the new scoring model was 0.792 (95% CI, 0.721—0.863). CI, confidence interval

ROC analysis for the scoring model indicated moderate discrimination with an AUC of 0.792 (95% CI, 0.721–0.863) (Fig. 1). The Hosmer–Lemeshow test indicated good calibration of this model (*P* = 0.716). The post hoc power analysis showed a power of 100% based on 151 patients at a 5% alpha level.

**Fig. 2:**
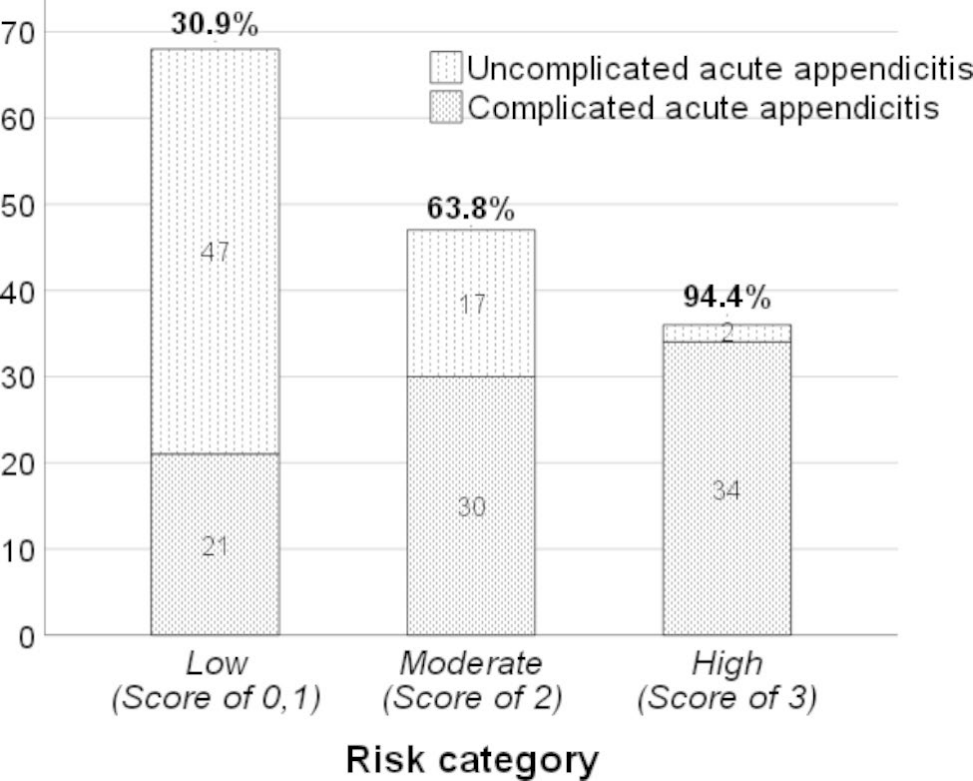
Risk category based on the probability of gangrenous/perforated appendicitis. The probability of gangrenous/perforated appendicitis in low, moderate, and high-risk categories were 30.9%, 63.8%, and 94.4%, respectively

The diagnostic performance of each score in the model is presented in Table 4. At a score of 2, the model had the best performance (maximum Youden’s index), and the sensitivity and specificity were 75.3% and 71.2%, respectively. The positive and negative likelihood ratios (LR) at scores of 1, 2, and 3 were 1.26 and 0.19, 2.62 and 0.35, and 13.2 and 0.62, respectively. For the clinical application, the scores were classified into three risk categories: low risk, 0–1; moderate risk, 2; and high risk, 3. The probability of GPA in each category was 30.9%, 63.8%, and 94.4% in the low-, moderate-, and high-risk categories, respectively (Fig. 2).

**Table 4:** Diagnostic Performance of Each Score in the Scoring Model for GPA

Score	Number of patients	Sensitivity (%)	Specificity (%)	LR+	LR-
0	20	100	0	1	-
1	48	95.3	24.2	1.26	0.19
2	47	75.3	71.2	2.62	0.35
3	36	40	97	13.2	0.62

GPA, gangrenous/perforated appendicitis; LR+, positive likelihood ratio; LR-, negative likelihood

## Discussion

In this study, we developed a novel scoring model based only on objective findings for predicting GPA in adults. We successfully identified three independent predictors for GPA using univariate and multivariate logistic regression analyses: CRP, the maximal outer diameter of the appendix, and the presence of appendiceal fecalith on the basis of objective, serological, or radiological findings. A scoring model was developed based on the regression coefficients assigned to these independent predictors. To the best of our knowledge, only a few studies have reported a scoring model based solely on objective findings for predicting GPA in adults.

In previous studies, various indicators have been reported to be predictors of GPA [13–21]. In this study, CRP as a serological marker and radiological findings such as the maximal outer diameter of the appendix and the presence of appendiceal fecalith were identified as independent predictors of gangrenous/perforated appendicitis; these results are consistent with those of previous studies [13–21].

Previously, we reported that the maximal outer diameter of the appendix and presence of appendiceal fecalith were associated with failure of non-operative management in UA [22]. We considered that the presence of fecalith leads to an ongoing obstruction with subsequent outflow obstruction of the lumen of the appendix. This increases the intraluminal pressure with an increase in inflammation. In addition, the diameter of the appendix has been considered to increase with an increase in the intraluminal pressure, which is reported to lead to the progression of inflammation and to the development of complicated appendicitis and early perforation [23]. Thus, we believe that a larger diameter of the appendix and the presence of appendiceal fecalith are strongly associated with GPA and the failure of non-operative management in UA.

CRP, synthesized primarily by the liver in response to inflammatory cytokines (interleukin-6, interleukin-8, and tumor necrosis factor-α), is one of the most frequently used inflammatory markers in clinical practice and is released following either an infection, inflammation, or tissue damage [24]. In acute infection, elevated CRP levels are generally associated with a high degree of inflammation [25], and the value of CRP reflects ongoing inflammation and/or tissue damage [24]. Therefore, we believe that elevated CRP levels are associated with GPA where there is ongoing inflammation. The level of elevated CRP corresponds to the time of the onset of inflammation and peaks around 48 h [24]. Several studies have reported that a longer duration of symptoms is associated with complicated acute appendicitis [14, 16, 17]. Taking these facts into consideration, we speculate that the increase in CRP levels reflects the time from the onset of inflammation and is associated with GPA.

In this study, we did not analyze variables of poor objectivity and reproducibility, such as the severity of pain, duration of symptoms, vital signs, and physical examination findings. Three variables in our scoring model, namely, CRP ≥ 7 mg/dL, the maximal outer diameter of the appendix ≥ 13 mm, and the presence of appendiceal fecalith are not only objective and reproducible, but can also be obtained with ease from routine laboratory tests and CT that are performed in most hospitals. The AUC of our new scoring model was 0.792 (95% CI, 0.721–0.863), indicating good diagnostic accuracy in predicting GPA. To validate our scoring model, we applied it to eligible patients who underwent emergency surgery in 2022 at our hospital. The resulting AUC was 0.782 (95%CI: 0.511-1.000), similar to the present study (data not shown). In addition, we examined two scoring models (including CT findings) from previous studies for comparison to the cases in our study using ROC analysis. The AUC of Ateme‘s model and Imaoka’s model were 0.792 (95%CI: 0.72–0.864) and 0.719 (95%CI: 0.637–0.801), respectively, with which our scoring model was comparable [14, 18].

Our scoring model may help in identifying patients with GPA in clinical practice. In addition, because of the objectivity and reproducibility of this model, it can be applied to patients who have difficulty with providing accurate clinical history or undergoing an abdominal examination, such as older people and those with impaired consciousness. At a score of 2, our model showed the best performance for the prediction of GPA, with the maximum Youden index. Moreover, a score of 1 had a sensitivity of 94% and a negative LR of 0.19; therefore, a score of 0 may be helpful in ruling out GPA appendicitis. Conversely, a score of 3 with a specificity of 97% and a positive LR of 13.2 may be helpful in diagnosing GPA. In risk stratification for clinical use, non-operative management may be selected in patients with low risk (a score of 0 and 1) under careful monitoring, whereas immediate appendectomy should be considered in those who are at high risk (a score of 3) of progressing to severe inflammation. With moderate risk (a score of 2), the treatment should be carefully selected, keeping in mind that non-operative management may not be successful.

This study had several limitations. Our study was a single-center retrospective study with a small sample size of Japanese patients. Additionally, our model has a risk of overfitting, and it has not been validated externally.　Thus, we consider that prospective studies with large sample sizes including different populations other than Japanese patients are needed to confirm the usefulness of our model and verify its applicability to a wide range of patients in clinical practice.

## Conclusion

In summary, we developed a novel scoring model for the prediction of GPA in adults by using objective and reproducible variables (CRP, maximal outer diameter of the appendix, and presence of appendiceal fecalith) that are readily available with ease from laboratory testing and CT scans. Thus, our scoring model can be easily used in clinical practice in most hospitals and even in difficult settings. Our scoring model may identify GPA with good diagnostic accuracy and help in judging the degree of urgency and deciding the plan of management.

## Data Availability

All data generated or analyzed during this study are included in this article. Further inquiries can be directed to the corresponding author.

## List of abbreviations

ROC: Receiver operating characteristic
CT: Computed tomography
GPA: Gangrenous/perforated appendicitis
UA: Uncomplicated appendicitis
CRP: C-reactive protein
AUC: Area under the curve
